# Non-Invasive Biomarkers for Early Detection of Breast Cancer

**DOI:** 10.3390/cancers12102767

**Published:** 2020-09-27

**Authors:** Jiawei Li, Xin Guan, Zhimin Fan, Lai-Ming Ching, Yan Li, Xiaojia Wang, Wen-Ming Cao, Dong-Xu Liu

**Affiliations:** 1 The Centre for Biomedical and Chemical Sciences, School of Science, Faculty of Health and Environmental Sciences, Auckland University of Technology, Auckland 1010, New Zealand; jiawei.li@aut.ac.nz (J.L.); xin.guan@aut.ac.nz (X.G.); yan.li@aut.ac.nz (Y.L.); 2 Department of Breast Surgery, the First Hospital of Jilin University, Jilin University, Changchun 130021, China; fanzm@jlu.edu.cn; 3 Auckland Cancer Society Research Centre, Faculty of Medical and Health Sciences, University of Auckland, Auckland 1023, New Zealand; l.ching@auckland.ac.nz; 4 Department of Breast Medical Oncology, Cancer Hospital of the University of Chinese Academy of Sciences, Zhejiang Cancer Hospital & Institute of Cancer and Basic Medicine (IBMC), Chinese Academy of Sciences, Hangzhou 310022, China; wangxj@zjcc.org.cn

**Keywords:** breast cancer, biomarker, diagnosis, detection, blood, body fluid

## Abstract

**Simple Summary:**

Early diagnosis of breast cancer greatly increases the chance of cure and survival from the disease. The mammogram is widely used for early detection of breast cancer, but its effectiveness and accuracy have been a concern for a long time as well as its inability in detecting small cancers, especially in women with dense breast tissues. Therefore, it is an unmet clinical need to develop a simple, convenient test to overcome the shortcomings of mammography. Liquid biopsy, which is based on the analysis of body fluids, has attracted much attention in the search for cancer biomarkers. Recent advances in analytical techniques have gradually made it possible to detect breast cancer early through a biomarker analysis of blood, nipple aspirate fluid, sweat, urine, tears, or the breath. We envision that a simple blood or breath test holds great promise as a biomarker for early detection of breast cancer in the near future.

**Abstract:**

Breast cancer is the most common cancer in women worldwide. Accurate early diagnosis of breast cancer is critical in the management of the disease. Although mammogram screening has been widely used for breast cancer screening, high false-positive and false-negative rates and radiation from mammography have always been a concern. Over the last 20 years, the emergence of “omics” strategies has resulted in significant advances in the search for non-invasive biomarkers for breast cancer diagnosis at an early stage. Circulating carcinoma antigens, circulating tumor cells, circulating cell-free tumor nucleic acids (DNA or RNA), circulating microRNAs, and circulating extracellular vesicles in the peripheral blood, nipple aspirate fluid, sweat, urine, and tears, as well as volatile organic compounds in the breath, have emerged as potential non-invasive diagnostic biomarkers to supplement current clinical approaches to earlier detection of breast cancer. In this review, we summarize the current progress of research in these areas.

## 1. Introduction

Breast cancer is the most commonly diagnosed cancer in women worldwide. The incidence and mortality rates for female breast cancer far exceeded those for other cancers [1]. Although the incidence rate of breast cancer has risen, the mortality rates have steadily fallen due to early diagnosis and better treatments [2]. Early detection of breast cancer often leads to better outcomes. According to Cancer Australia’s National Cancer Control Indicators, the relative survival for females diagnosed with early-stage breast cancers at diagnosis was much higher than that for those with advanced breast cancers. Survival for early-stage breast cancer (stage 1) remained at 100% at 1, 3, and 5 years from diagnosis. However, survival for metastatic breast cancer (stage 4) reduced to 69% at 1 year, 47% at 3 years, and 32% at 5 years from diagnosis [3]. Therefore, detection of breast cancer at an early stage plays a pivotal role in reducing the mortality.

Mammogram screening has been commonly used for early detection of breast cancer in many high-income countries. However, the benefits and limitations of mammography have been a heated international debate for decades [4,5,6,7,8,9]. The main concerns with mammography are radiation and overdiagnosis. Studies have observed that the X-rays used for mammography could be a contributor to the onset of cancer. Overdiagnosis through breast cancer mammogram screening has been recognized as the most important adverse event and the estimates of overdiagnosis range from 0% to 40–50% depending on invitational age and methods [10,11]. In addition, mammograms cannot detect small tumors and are less accurate in cancer detection in women with dense breasts. Therefore, it is imperative to have alternative tools for early detection of breast cancer [12].

**Figure 1:**
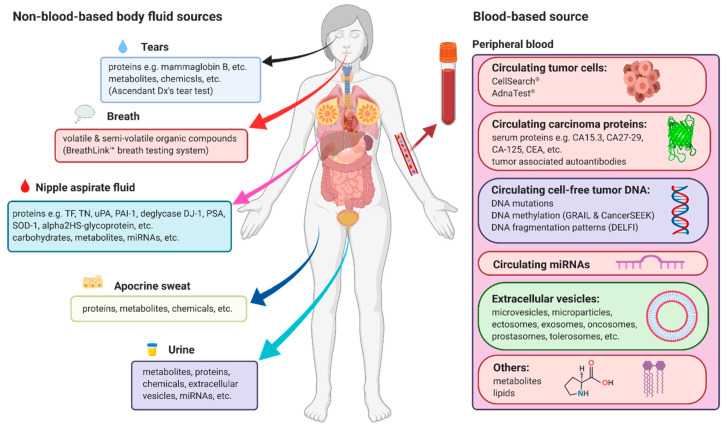
Overview of current sources and main measurement types of non-invasive biomarkers for early detection of breast cancer. Figures were created with BioRender.com (https://biorender.com/).

Breast cancer diagnosis is usually confirmed by using needle or surgical biopsy, which is not only invasive but also not necessary in most cases when tumors are benign. Therefore, much attention and many research efforts have been focused on the development of non-invasive and more convenient biomarkers that allow earlier detection of breast cancer. It has been reported that non-invasive body fluid-based tests, including circulating carcinoma antigens (CAs), circulating tumor cells (CTCs), circulating cell-free tumor nucleic acids (DNA or RNA), circulating microRNAs (miRNAs), circulating extracellular vesicles (EVs) in the peripheral blood, nipple aspirate fluid (NAF), sweat, urine, and tears, as well as volatile organic compounds (VOCs) in exhaled breath, have the potential to supplement current clinical approaches to earlier detection of breast cancer (Figure 1) [13,14]. In this review, we summarize the current progress of research in these areas (Table 1).

## 2. Blood-Based Biomarkers

Compared with imaging and biopsy cancer detection approaches, blood tests are not only convenient and non-invasive (or minimally invasive), but also widely acceptable, readily reproducible and cost effective. Cancer cells often produce specific proteins, nucleic acids or other cellular vesicles, and shed live cells or dead cell debris into the blood. Analyzing for the existence of those components in the blood may provide a method of detection of the cancer.

### 2.1. Circulating Carcinoma Proteins

Circulating carcinoma proteins are associated with proliferation, invasion, metastasis, aggressiveness, angiogenesis, oncogenic signaling, and immune regulation of tumor cells. Thus, they have the potential to serve as markers for cancer detection [62]. A number of serum carcinoma protein markers in breast cancer have been identified, including CA15-3, CA27-29, CA-125, carcinoembryonic antigen (CEA), tissue polypeptide antigen (TPA), circulating extracellular domain of human epidermal growth factor receptor 2 (HER2), and tissue polypeptide-specific antigen (TPS) [63,64]. These markers are mainly used in monitoring response to therapy in patients with advanced disease and none of them has been used alone for screening because of low diagnostic sensitivity for early disease or lack of specificity [65,66]. Although a combination of several such biomarkers may increase the sensitivity and specificity, a study demonstrated that no combination of the selected ten breast cancer serum protein markers, including CA15-3, CA125, and CEA, could accurately predict early-stage breast cancer [67]. However, when combined with tumor-associated autoantibodies, serum protein biomarkers were able to achieve 81.0% sensitivity and 78.8% specificity for detection of breast cancer with an area under the receiver operating characteristic (ROC) curve (AUC) of 0.89 [15]. Combined with clinical patient characteristics, a combinatorial serum biomarker panel, Videssa Breast (Table 1), could achieve a comprehensive 93% sensitivity and 98% negative predictive value for women aged 25–75 [16], and a 99.1% negative predictive value, 87.5% sensitivity, and 83.8% specificity for women under age 50 [17]. However, in one study, the negative predictive value of the Videssa Breast liquid biopsy was not high enough to defer tissue biopsy, and its specificity was also too low to help predict results in high-risk solid lesions [68]. Thus, although Videssa Breast tests, in combination with breast imaging results, could improve the accuracy and reduce the false-positive rate of breast cancer detection in women aged over 50 compared with mammography alone, further improvements of detection sensitivity by using new technologies are still needed [69,70].

Of note, other secreted oncoproteins, such as TFF1 [71], TFF3 [72], ARTN [73], and SHON [74,75], have been described in breast cancer. Whether these proteins could be used for early detection of breast cancer remains to be investigated.

### 2.2. Circulating Tumor Cells

Tumor cells may enter into the peripheral blood of cancer patients either through active intravasation or passive shedding from the primary or metastatic tumors. The presence of CTCs in early-stage breast cancer increases the risk of recurrence and death [76,77]. However, the measurement of CTCs has not been recommended for breast cancer diagnosis because of low sensitivity and reproducibility [64]. CTCs are rare and there is as few as one CTC per billion normal blood cells [78]. Many technologies have been assessed for enumeration and analyses of CTCs based on physical characteristics, immunomagnetic separation, and immunofluorescence/enzyme-linked immunosorbent assays, as well as reverse transcription polymerase chain reaction (RT-PCR) assays [78,79,80,81]. Among the many CTC technologies, CellSearch^®^ (Janssen Diagnostics, Raritan, NJ, USA) and AdnaTest^®^ (AdnaGen AG, Langenhagen, Germany) have been widely studied in clinical trials. The CellSearch^®^ system is a semiautomated antibody-based assay based on immunofluorescence and flow cytometry. CTCs are enriched using epithelial cell adhesion molecule (EpCAM) antibodies, and counted by cytokeratin positivity, positive nuclear staining, and CD45 negativity. The AdnaTest^®^ assay is an RT-PCR based test for detecting cancer-specific mRNA markers, e.g., GA733-2, MUC1, and HER2 for breast cancer, after immunomagnetic enrichment of tumor cells with MUC1 and EpCAM antibodies. Both assays have a similar detection sensitivity in detecting two or more CTCs, but their overall positive agreement was only 73% for CTC ≥ 2 and 69% for CTC ≥ 5, respectively [18]. However, the CellSearch^®^ system was shown to be superior to the AdnaTest^®^ assay in predicting clinical outcome in advanced breast cancer [82], while the AdnaTest^®^ assay was superior to CellSearch^®^ system in terms of overall detection rates [83]. Nevertheless, the CellSearch^®^ system is currently the only FDA-approved CTC assessment for detecting and enumerating CTCs of metastatic breast cancer for prognosis. The presence of CTCs above a cut-off level—five cells per 7.5 mL blood—is associated with shorter survival, which makes CTCs a useful prognostic biomarker [84,85]. However, the low frequency of CTCs, together with the heterogeneity of antigens expressed on the surface of CTCs, makes the detection very difficult and limits their value as a diagnostic tool for patients with early-stage breast cancer. Therefore, more sensitive technologies to target a broad range and variety of CTCs are still needed [86].

### 2.3. Circulating Cell-Free Tumor DNA

Circulating tumor DNA (ctDNA) in the bloodstream has emerged as a promising biomarker of disease status for breast cancer [20,87,88,89,90]. An elevated level of ctDNA has been found to be associated with advanced-stage breast cancer and metastasis [91,92]. Breast cancer patients had significantly higher levels of ctDNA than healthy controls [91]. The ctDNA concentrations at levels ≥0.75% could be detected in breast cancer patients with a sensitivity of >90% and a specificity of >99% [19]. Some ctDNA was also detected in 97% (29/30) women with metastatic breast cancer [20]. It has also been demonstrated that analyses of mutations in ctDNA could detect early-stage tumors [93]. For example, ctDNA assays targeting known tumor mutations using droplet digital PCR (ddPCR) assays demonstrated a sensitivity of 93.3% and a specificity of 100% in detection of early-stage breast cancer [21]. Similarly, TP53 mutations detected in ctDNA may be useful for breast cancer screening for those with BRCA1 mutations [94]. Chromosome instability is one of the hallmarks of tumors. We have shown that chromosome instability analysis of cell-free DNA (cfDNA) using low-pass whole-genome sequencing can detect breast cancer recurrence more accurately than traditional serum CA15-3 and CEA biomarkers [90]. Another study also shows that amplification of chromosome 1q21.3 detected in ctDNA from blood is significantly associated with breast cancer early relapse [95].

**Figure 2:**
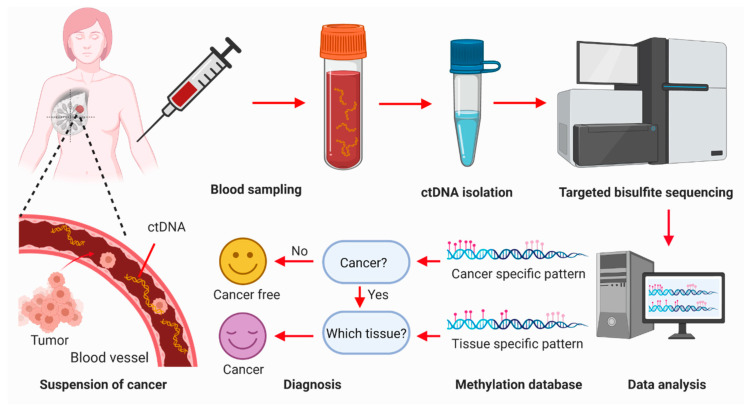
Diagram of the procedures of breast cancer detection through methylation patterns of circulating cell-free DNA in the blood. Breast tumor and normal tissue shed DNA fragments into the blood. Peripheral blood is withdrawn from human subjects and circulating tumor DNA (*ctDNA*) is isolated from the blood sample. The methylation status of the ctDNA is determined by targeted bisulfite methylation sequencing. The methylation patterns of the ctDNA fragments are analyzed. The presence or absence of cancer cells is thus determined by comparing the methylation patterns with those in the methylation database, which is constructed from those individuals with and without cancer. The origin of cancer is determined using unique tissue-specific methylation patterns. Figures were created with BioRender.com (https://biorender.com/).

DNA methylation alteration has been frequently observed in cancer. A number of genes, such as p16 [96], BRCA1 [97,98], RASSF1A [22,23], APC [99], and GSTP1 [100], are reported to be hypermethylated in breast cancer. Technological advances in epigenetics have opened an era to detect breast cancer using methylation signatures of cfDNA [101,102,103]. Although the sensitivity of a single gene methylation is too low to be used for early screening of breast cancer [22,99,104], a panel of epigenetic markers can greatly increase the sensitivity required for breast cancer detection [105,106]. For example, the methylation patterns of six genes in cfDNA achieved 79.6% sensitivity and 72.4% specificity in the diagnosis of breast cancer (*n* = 749) (Table 1) [24]. The methylation analysis of a panel of 16 genes, including 12 novel epigenetic markers and four internal control markers, could discriminate cancer patients from normal healthy controls with a sensitivity of 86.2% and a specificity of 82.7% (Table 1) [25]. Of note, a targeted methylation panel covering 103,456 distinct DNA regions (17.2 Mb) and 1,116,720 CpGs was developed by GRAIL Inc (Menlo Park, CA, USA) (Figure 2). In a prospective case–control sub-study of 6689 participants (2482 cancer of > 50 cancer types and 4207 non-cancer), the GRAIL DNA methylation patterns of cfDNA can detect various types of cancers at 99.8% specificity (training set: N = 844) or 99.3% specificity (validation set: N = 359) with a <1% false-positive rate [26]. The precision for breast cancer was 96% (82/85) for training and 93% (40/43) for validation [26].

Except methylations, fragmentation patterns of cfDNA could also be used for early detection of breast cancer. An approach called “DNA evaluation of fragments for early interception” (DELFI) was developed to detect a large number of abnormalities in cfDNA by genome-wide analysis of fragmentation patterns [27]. DELFI profiles of cfDNA could detect breast cancer at a sensitivity of 57% (31/54) and a specificity of 98%. When DELFI fragmentation patterns of cfDNA are combined with mutations detected in cfDNA [93], the detection sensitivity increased to 65% (35/54 patients) with a specificity of 98% [27].

Compared with carcinoma proteins and CTCs, ctDNA has several advantages, being a better and more sensitive marker for monitoring tumor burden [107,108]. It has a greater dynamic range representing the heterogenetic features of tumors, a shorter half-life, and is more specific to malignancy [109]. Moreover, ctDNA mutation and methylation assays are able to identify the presence of relatively early cancers as well as localize the organ of origin of cancers [38]. However, the application of ctDNA as a non-invasive diagnostic clinical biomarker still faces several technical issues. Most cfDNA is released by normal cells into the circulation as a result of cell death. The amount of ctDNA is very low, highly variable and comprises <0.1% of the total cfDNA, particularly in patients with early-stage cancers [110], making detection of the ctDNA challenging [107]. The lower copy numbers of ctDNA compared with those of wild-type cfDNA and the limited accuracy of current sequencing technologies contribute to the limitation in ctDNA detection [111]. Therefore, a highly sensitive technique to detect ctDNA is still required. For example, the CRISPR-based diagnostic platform may be used to identify mutations in cell-free tumor DNA with high sensitivity and specificity [112].

### 2.4. Circulating miRNAs

The miRNAs are small regulatory RNA molecules that mediate target mRNA expression by base pairing to complementary sequences in the 3′ untranslated region [113]. It was shown that circulation miRNAs in the serum and plasma of patients could distinguish patients with prostate cancer from healthy controls [114]. In breast cancer, a large number of miRNAs have been observed to be significantly upregulated in the plasma of patients, although a small number of miRNAs were found to be downregulated when compared to healthy controls [115,116]. Studies have demonstrated that a combination of certain circulating miRNAs were able to distinguish breast cancer from normal and healthy controls, as well as differentiate breast cancer from benign lesions [28,29,117,118,119,120,121,122]. For example, a panel of five miRNAs could detect breast cancer with a sensitivity of 97.3%, a specificity of 82.9%, and an accuracy of 89.7% (Table 1) [28]. Another panel of seven miRNAs could differentiate triple-negative breast cancer from healthy women with an accuracy of 79%, a specificity of 74.2%, and a sensitivity of 83.8% (Table 1) [29]. Therefore, differentially expressed circulating miRNAs are potential diagnostic biomarkers for breast cancer detection [14,122,123]. However, there is little consistency among the circulating miRNA panels identified in different studies. So far, there are no panels of circulating miRNAs that are ready for breast cancer diagnosis in a clinical setting [122].

### 2.5. Extracellular Vesicles

The term EV is a generic term used to refer to all types of vesicles that exist in the extracellular space, including microvesicles, microparticles, ectosomes, exosomes, oncosomes, prostasomes, and tolerosomes [124,125]. EVs, secreted from normal and cancer cells, are a complex of lipids, proteins, DNAs, various RNAs, and other biomolecules enclosed by a lipid bilayer with transmembrane proteins [126]. Because EVs can mirror the features of the origin and the state of the tumor, they have gained increasing attention as cancer biomarkers. An elevated number of EVs has been found in the peripheral blood of breast cancer patients compared with healthy controls [127,128,129,130,131]. However, the number of EVs alone is not specific enough for cancer diagnosis [132].

As an alternative to EV count, their molecular cargos may be a better cancer biomarker, especially if the enclosed contents are cancer related, such as amplified oncogenes and oncoproteins [133,134]. For example, the levels of cancer-associated fibronectin and developmental endothelial locus-1 (Del-1) proteins detected in circulating EVs were significantly elevated at all stages of breast cancer, and returned to normal after tumor removal [30,31]. Similarly, focal adhesion kinase (FAK), epidermal growth factor receptor proteins [130], survivin [128], EMMPRIN [135], and various miRNAs [136] were also significantly enriched in EVs isolated from the plasma of breast cancer patients. Moreover, compared with healthy controls, specific miRNAs that were overexpressed in breast cancer sera were enriched in EVs [136]. Thus, analyzing cancer-related contents enclosed in EVs could help early-stage breast cancer diagnosis and distinguish breast cancer from benign and noncancerous diseases. Such analysis could provide a diagnostic tool with higher sensitivity and specificity compared with whole-blood analysis, as cancer-derived EVs preserve molecules that are relevant to diagnosis [126,132].

EVs possess great potential as novel non-invasive diagnostic molecular biomarkers of breast cancer. However, there are still issues in how to identify and isolate EVs, such as contamination with cells and platelet remnants [137]. There are many technologies that have been used to isolate and characterize EVs [138,139,140,141,142]. Currently, there are no standardized methods of isolation and quantification of EVs. It has been observed that the profiles of EVs captured from the same source of materials are dependent on isolation methods used [142]. This has made the use of EVs as a diagnostic biomarker more challenging. Optimization and standardization of the methods and protocols of EV isolation and purification are urgently required [141,142]. Moreover, our knowledge of EVs is still limited. The precise molecular mechanisms of biogenesis, release, and functions of EVs also remain to be investigated [143].

### 2.6. Other Emerging Blood-Based Biomarkers

Metabolite profiles in the blood are also potential biomarkers to differentiate primary breast cancer patients from healthy controls. A panel of seven metabolites (Glu, Orn, Thr, Trp, Met-SO, C2, and C3) in the plasma could detect breast cancer with an AUC of 0.87 in the training cohort (80 cancer vs. 100 healthy controls) and an AUC of 0.80 in the validation cohort (109 cancer vs. 50 healthy controls) [32]. The expression levels of three metabolites (8-hydroxy-2ʹ-deoxyguanosine, 1-methylguanosine, and 1-methyl adenosine), together with CA15-3, could achieve 88.8% sensitivity and 86.8% specificity in the detection of early-stage breast cancer based on a serum metabolome score [33]. In a prospective case–control study, the signature of seven metabolites (dimethyldodecane, galactose, α-glyceryl stearate, methyl stearate, 1(1-methoxycarbonyethyl)-4-(2-methyl-2-trimethylsilyl-oxypropyl) benzene, tetradecane, and glucopyranoside) in serum revealed a distinct separation between healthy controls and breast cancer patients at a sensitivity of 96% and a specificity of 100% [34]. Four plasma metabolites (L-octanoylcarnitine, 5-oxoproline, hypoxanthine, and docosahexaenoic acid) were also identified as potential biomarkers for diagnosis of breast cancer, with L-octanoylcarnitine showing a 100.0% positive predictive value [35]. Interestingly, one study identified metabolites, which included four knowns (caproic acid, taurine, stearamide and linoleic acid) and five unknowns (C26H43ClN4S3, C26H51N5O4, C9H16O3S, C23H30N2S, and 278.1552@9.641), in the plasma that could distinguish between breast cancer patients and healthy controls, irrespective of the breast cancer type, at a high sensitivity and specificity up to 100% with high accuracy [36]. However, these need to be validated.

One study identified 18 serum free fatty acids from serum samples of 16 breast cancer patients and 18 breast adenosis patients. Five candidates (palmitic acid, oleic acid, cis-8,11,14-eicosatrienoic acid, docosanoic acid, and the ratio of oleic acid to stearic acid) were shown to be potential serum biomarkers for differential diagnosis of breast cancer [144]. A panel of five serum free fatty acids (C16:1, C18:3, C18:2, C20:4, and C22:6) was shown to possess the ability to differentiate early-stage breast cancer patients (*n* = 140) from healthy controls (*n* = 202), with an AUC of 0.953, a sensitivity of 83.3%, and a specificity of 87.1% [37].

### 2.7. Multi-Analyte Blood Tests

Multi-analyte blood tests combine the detection advantages of several blood marker assays for cancer detection and localization, thus improving the accuracy. The CancerSEEK test is such a multi-analyte test that evaluates the levels of eight circulating protein markers and the presence of mutations in 16 genes in cfDNA in the blood (Table 1) [38]. CancerSEEK has a median sensitivity of 70% and a specificity of >99% in identifying patients with one of the eight cancer types evaluated [38]. However, the cancer detection effectiveness of CancerSEEK ranges from 98% for ovarian cancer to only 33% for breast cancer. A feasibility study of CancerSEEK tests has been conducted on a general population of 10,000 women with no history of cancer [39]. The blood test detected 26 previously unknown cancers of different types with a sensitivity (26/96) of 27.1%, a specificity of 98.9% (9707/9815), and a positive predictive value of 19.4% (26/134) [39]. Among the participants, 27 breast cancers were identified. Among them, only one breast cancer was first detected by the blood test, 20 by standard-of-care screening and six by other imaging means [39]. Therefore, CancerSEEK tests only have a low sensitivity of 3.7% (1/27) in breast cancer detection.

The CancerSEEK test was developed from a case–control study, which introduced a possible bias in the utility of the assay [145,146]. Although a remolding of the CancerSEEK test, e.g., the CancerA1DE method, increased the sensitivity to 70% for breast cancer detection at a 99% specificity [40], it is unlikely that the CancerSEEK test will be effective in breast cancer detection [39].

## 3. Non-Blood-Based Biomarkers

Besides blood, other body liquids, including urine, NAF, tears, and sweat, as well as the breath of patients, have also been investigated for breast cancer diagnosis.

### 3.1. Biomarkers in Urine

Human urine is one of the most useful body biofluids for routine testing. A number of studies have suggested that urine could contain potential biomarkers for breast cancer screening, ranging from metabolome profiling and proteomic profiling to exosomic analysis [147,148,149,150].

Phospholipids are building blocks for cellular membranes. Phosphatidylcholine (PC), phosphatidylethanolamine (PE), and sphingomyelin are the most abundant phospholipids and comprise up to 80% of the membrane. Increased phospholipid metabolism, particularly PC and PE or their precursor molecules, has been observed in breast cancer tissues [151,152]. Loss of the wild-type BRCA1 allele has been shown to increase lipid production in breast cancer cells [153]. Using nanoflow liquid chromatography/electrospray ionization tandem mass spectrometry, the urine samples of patients with breast cancer, both before and after surgery, were qualitatively and quantitatively analyzed [154]. In that analysis, twenty-one PCs and 12 PEs were identified in the patient samples, and two PCs (16:0/16:0 and 14:1/20:4) and one PE (18:3/18:0) were not detectable after surgery. Moreover, compared with the controls, the total concentrations of PCs and PEs of patient urine samples increased by 44% and 71%, respectively, but significantly decreased after surgery [154].

One study has demonstrated that 59 urinary proteins are differentially detected (>3-fold change) in breast cancer patients compared with healthy control individuals, and of them, 36 urinary proteins are stage specific [155]. A panel of 13 upregulated proteins could be used for breast cancer detection. The panel consists of leucine LRC36, MAST4, DYH8, HBA, PEPA, filaggrin, MMRN2, AGRIN, NEGR1, FIBA, keratin KIC10, and two uncharacterized proteins, C4orf14 (CD014) and CI131 [155]. These stage-specific markers are associated with pre-invasive breast cancer in ductal carcinoma in situ (DCIS), early invasive breast cancer, and metastatic breast cancer [155].

It was demonstrated that the urinary miRNA profile of primary breast cancer patients was different from that of healthy controls. The urinary miRNA profiles could separate the patients from healthy controls with a sensitivity and specificity of 91.7% and an AUC of 0.932 [41]. A study explored the diagnostic potential of urinary exosomal miRNAs in a case–control study of 69 breast cancer patients and 40 healthy controls [42]. They identified a specific panel of four urinary miRNA types (miR-424, miR-423, miR-660, and let7-i) that could discriminate breast cancer patients from healthy controls with a sensitivity of 98.6% and a specificity of 100% (Table 1) [42].

Urine is also a source of exosomes. One study isolated urine exosomes from patients with breast cancer and 26 healthy females and determined the expression of miRNA-21 and matrix metalloproteinase-1 (MMP-1) in the isolated exosomes by quantitative RT-PCR. They discovered that the expression of urine exosome-derived miRNA-21 and MMP-1 could be diagnostic markers for cancer detection with a combined sensitivity of 95% and a specificity of 79% [43].

Many studies have analyzed the metabolomic and proteomic profiles using technologies such as NMR spectroscopy [150,156], gas chromatography/mass spectrometry [48], liquid chromatography coupled to mass spectrometry [157,158,159,160], and capillary electrophoresis coupled to mass spectrometry [161]. Based on these profiles, they identified cancer-specific patterns and used calculated models for early diagnosis of breast cancer. For example, the combination of succinic acid and dimethyl-heptanoylcarnitine were able to separate breast cancer patients (*n* = 31) from healthy controls (*n* = 29) with a sensitivity of 93.5% and a specificity of 86.2% [44].

Because urine collection is non-invasive and convenient, it has been used for the discovery of cancer biomarkers. Changes in proteins, metabolites, miRNAs, or other cellular components in urine could potentially indicate the presence of breast cancer. However, current reported urinary breast cancer biomarkers are still in the biomarker discovery phase, and their specificity and sensitivity have to be validated in cohort studies [162].

### 3.2. Volatile Biomarkers in the Breath

Human breath contains VOCs and semi-volatile compounds that are released by the body as a result of normal metabolic activity or due to pathological disorders. The presence of cancer cells can affect both the identity and abundance of these compounds in the exhaled breath of cancer patients. Chemical analyses of exhaled breath from patients have been exploited for diagnosing many different types of cancers [163,164].

In the 1990s, the use of breath testing for breast cancer was demonstrated with the finding that women with breast cancer had an increased level of pentane in their breath [165]. It was soon found that a set of VOC markers of oxidative stress in the breath could distinguish breast cancer patients from healthy volunteers with a sensitivity of 94.1% (48/51) and a specificity of 73.8% (31/42) [45]. More importantly, this breath test accurately identified women with breast cancer with a negative predictive value superior to a screening mammogram test, although the positive predictive value was lower than that of a screening mammogram test [45]. This clearly suggested that breath tests could be employed as a primary screen for breast cancer.

The breath samples of patients with breast cancer and pair-matched healthy controls were analyzed in a prospective study by gas chromatography/mass spectrometry [46]. A total of 109 different VOCs was identified in the breath of breast cancer patients and controls. Of them, five specific VOCs, 3-methylhexane, decene, caryophyllene, naphthalene, and trichlorethylene in the breath, could distinguish those with breast cancer from healthy controls [46]. In another study, a fuzzy logic model was constructed with five breath VOC biomarkers, 2-propanol, 2,3-dihydro-1-phenyl-4(1H)-quinazolinone, 1-phenyl-ethanone, heptanal, and isopropyl myristate in the training set (*n* = 64); and the model could predict breast cancer with 93.8% sensitivity and 84.6% specificity in the test set (*n* = 29) [47]. More importantly, the same model showed a negative prediction of breast cancer for 16 out of 50 (32.0%) women with abnormal mammograms and their cancer-free prediction was confirmed by biopsy [47].

The ability of a breath test for breast cancer detection has been further demonstrated in a number of other studies [166,167,168,169]. With improvements in breath collection methods, analytic techniques and data modeling, a clinical breath test for breast cancer is being actively developed. One study used inexpensive commercial electronic noses to identify unique breath patterns in women with breast cancer and an average of 95% accuracy in the classification of breast cancer patients was achieved through an artificial neural network model based on the data obtained from the electronic noses [48]. Menssana Research Inc. (Fort Lee, NJ, USA) has developed BreathLink™ point-of-care breath testing systems for the collection, concentration, and analysis of VOCs in human breath to detect breast cancer in women. A pilot study showed that this system could accurately identify women with breast cancer and abnormal mammograms [169]. The results of this prospective clinical validation study were updated recently [49]. To facilitate the collection of breath and analysis of VOCs, ultra-clean breath collection balloons have been developed by Menssana Research Inc. The balloons were used to collect breath samples from 54 female breast cancer patients and 124 cancer-free controls. Breath VOC biomarkers of breast cancer identified by a Monte Carlo analysis in the training set were incorporated into a multivariate algorithm to predict disease in the validation set to generate a discriminant function (DF) value from the predictive algorithms [50]. Breast cancer risks can be accurately predicted based on the DF scores, with a low DF value indicating a low risk of breast cancer at a negative predictive value of > 99.9% and a high DF value indicating a high risk of breast cancer at a positive predictive value rising to 100% [50].

Breath VOC tests are certainly a very promising approach to screening women for breast cancer. Breath tests are non-invasive, painless, completely safe, and cost-effective. They could potentially reduce the use of mammograms in clinics for the screening and monitoring of breast cancer. However, a number of factors, including the breath collection methods, patient’s physiologic conditions, test environments, and methods of analysis, will affect the accuracy of VOC breath test results, and standardized procedures are still required [164].

### 3.3. Biomarkers in NAF

NAF is a natural secretion produced in the breasts by breast epithelial duct cells. It can be collected by nipple aspiration or by other methods in healthy nonlactating women. The color of NAF and the presence of specific biomarkers in NAF could be used to diagnose breast cancer. It has been observed that women with bloody or brown nipple discharge suffer a higher risk of breast cancer than those with white, cream, green, or yellow nipple discharge [170]. NAF contains high concentrations of proteins, carbohydrates and metabolites, such as amino acids, organic acids, and fatty acids. Several studies have suggested that the levels of drug, protein, and hormone levels in NAF more closely reflect the products of breast tissue metabolism and exposures than those in plasma or serum [171]. A study analyzed NAFs collected from healthy women using both nuclear magnetic resonance and gas chromatography/mass spectrometry [171]. In that study, a total of 38 metabolites, including amino acids, organic acids, fatty acids, and carbohydrates, were identified by the two analytical techniques. Eight metabolites are unique to NAF, 19 unique to plasma, and 24 are shared metabolites, indicating that the metabolic profile of NAF is distinct from that of matched plasma samples [171]. In addition, NAF also contains exfoliated breast epithelial cells, from which breast cancer originates [172]. Therefore, NAF is arguably a better source than other body fluids for the discovery of biomarkers for breast cancer.

The Thomsen–Freidenreich (TF) antigen and its biosynthetic precursor, Tn antigen, are often aberrantly glycosylated in cancer cells. A study examined the expression of TF and Tn in NAF samples from 25 breasts with cancer and 25 normal breasts [51]. Of the 25 NAF samples from breasts with cancer, 19 samples had TF and 20 samples had Tn, whereas only one of the 25 NAF samples from breasts without cancer contained Tn and neither TF nor Tn was detected in the rest, indicating that TF and Tn can be used as detection biomarkers for breast cancer [51]. A direct immunoassay of TF expression levels in NAFs of 124 women requiring biopsy because of a suspicious breast lesion demonstrated that TF concentrations in NAFs could distinguish women with precancer and cancer from those with benign disease [52]. Urinary plasminogen activator (uPA) and its inhibitor PAI-1 have been shown to promote tumor invasion and metastases by regulating the degradation of the extracellular matrix. Compared with women with benign disease, women with atypia and cancer were found to have higher concentrations of uPA and PAI-1 in NAFs [53]. In combination with TF, uPA could predict breast cancer in both pre- and post-menopausal women with 84-92% accuracy [53]. When TF, uPA, and PAI-1 were all combined, the predictive ability reached 100% in the cohort studied [53]. With a cut-off level of 3.0 ng/mL, the protein deglycase DJ-1 in NAFs could detect the presence of breast cancer with 85.9% specificity and 75% sensitivity [54].

High prostate-specific antigen (PSA) levels have been widely used as a diagnostic biomarker for prostate cancer. However, high levels of PSA in NAFs were found in all women with no risk factors or 90% of those with a family history of breast cancer, whereas women with precancerous or invasive cancer had reduced levels of PSA [173,174]. Such an inverse correlation was also found with the superoxide dismutase SOD-1. Breast cancer patients had a lower concentration of SOD-1 in their NAFs compared with healthy individuals [175].

Proteomic analyses of NAFs have been conducted to screen for diagnostic markers for breast cancer. One study analyzed paired NAFs of 18 women with low invasive breast carcinoma (stage I or II) and four healthy controls and identified 39 differentially expressed proteins between tumor-bearing and disease-free breasts [55]. Tumor-bearing breasts overexpressed lipophilin B, beta-globin, hemopexin, and vitamin D-binding protein precursor, but underexpressed alpha2HS-glycoprotein [55]. Higher dehydroepiandrosterone (DHEA) concentrations in NAFs were associated with breast cancer, especially estrogen receptor-positive cases [56].

NAF is also a source of miRNAs and contains more miRNA species than serum [176]. It was found that miR-3646 and miR-4484 were upregulated while miR-4732-5p was downregulated in the NAFs of patients with breast cancer, compared with those with benign breast lesions [177]. Therefore, miRNA analyses of NAFs are potentially very useful for the detection of breast cancer [178,179]. More studies comparing miRNA profiles of cancerous and healthy NAFs are underway [176].

Despite the low volume of samples, NAF collection is simple, quick, reliable, and reproducible [55]. Moreover, analytical reproducibility of NAF samples was high across different extraction and analysis days [171]. Therefore, NAF is an ideal source of biomarkers for early diagnosis of breast cancer [55]. Furthermore, because NAF is derived directly from the breast ductal system, future characterization of NAF may provide valuable, more specific, and sensitive information on breast cancer diagnosis and treatment [176]. However, in order to provide an accurate screening approach for breast cancer-specific biomarkers, increased success in NAF sample collection methods, normalization of the volume of the sample, and standardization of the analysis will be required.

### 3.4. Biomarkers in Tears

Tears are mainly produced by the lacrimal glands underneath the skin of the upper eyelids through filtration from blood plasma, which circulates in the organs and tissues of our body. Therefore, tears have been used as a source for external drug screening and pharmacokinetic studies, as well as for biomarker discovery of many diseases, including cancer [180].

Mammaglobin B (also called SCGB2A1 or lacryglobin) is overexpressed in breast cancer and serves as a biomarker for axillary lymph node micrometastases in breast cancer patients [181,182]. Mammaglobin B was first discovered in normal human reflex tears [183]. However, it is also detected in the tears of 88% of breast cancer patients [184]. A study analyzed the proteomic profiles of tears (and blood) of 10 breast cancer patients and 10 healthy controls using surface-enhanced laser desorption/ionization-time-of-flight mass spectroscopy and distinct protein expression profiles were identified [57]. This study demonstrated that breast cancer patients could be distinguished from controls based on a proteomic pattern in tear (and serum) fluid with a specificity and sensitivity of approximately 90% [57]. A further analysis of a larger cohort of breast cancer patients (*n* = 50) and healthy controls (*n* = 50) identified a panel of 20 differentiating protein biomarkers in tears, which could separate breast cancer patients from healthy controls with a specificity and sensitivity of approximately 70% [58]. Using a semi-quantitative method, more than 20 proteins were found to be differentially expressed in the tears of 25 patients with primary invasive breast carcinoma and 25 age-matched healthy controls [59]. Taken together, these studies have clearly indicated that tears are a source for potential non-invasive biomarkers for breast cancer diagnosis.

A tear protein-based breast cancer screening test is currently under development by Ascendant Dx, a diagnostic company. It was claimed to have up to 90% accuracy, implying a higher sensitivity than a mammogram [185].

Compared with blood, tears as a source of biomarkers have advantages. Tear sample collection is less invasive. Tears can be easily obtained from the surface of the eyes inside or outside the clinic for rapid and continuous monitoring of health. No prior protein filtration before analysis is needed as tears contain few solid proteins such as albumin. However, one particular analytical challenge is the much lower concentration of some molecules in tears compared with blood. Therefore, a sensitive and reliable screening technique is required for reliable and reproducible detection and quantification. More recently, a label-free surface-enhanced Raman spectroscopy biosensor has been designed for detecting or predicting breast cancer from human tears. It is a highly sensitive nanoplasmonic biosensor and allows for a rapid quantitative and qualitative analysis of tears on-site using a portable Raman spectrometer. Based on the preprocessed surface-enhanced Raman scattering (SERS) spectra of tear fluid obtained from five patients with breast cancer and five healthy controls, breast cancer can be predicted with a sensitivity of 92% and a specificity of 100% [60].

### 3.5. Biomarkers in Apocrine Sweat

Sweat is one of the less used non-invasively collectable biofluids for the discovery of biomarkers. Interests in screening for sweat biomarkers have been increasing with the advance in proteomic and metabolomic technologies. The composition of sweat is highly dynamic and alters significantly in various skin and other disorders. An in-depth profiling of sweat has identified 861 unique proteins and 32,818 endogenous peptides [186]. Sweat chloride tests have long been used clinically to diagnose cystic fibrosis [187]. There are reports that use sweat testing for drugs of abuse [188] and tuberculosis diagnosis [189]. A metabolite analysis of the sweat of patients with lung cancer versus smokers as control individuals has identified a panel of five metabolites that can detect lung cancer with 80% specificity and 79% sensitivity [190]. A similar sweat test for breast cancer has demonstrated that a mathematical–statistical model of 20 sweat markers was able to detect breast cancer with 97% sensitivity and 72% specificity [61]. However, these sweat-based cancer tests have not been clinically validated.

## 4. Conclusions

Over the last few decades, a lot of non-invasive or minimally invasive methods for early detection of breast cancer have been described to overcome the limitations of the current mammogram screening. Blood, other body fluids, and breath all contain potential biomarkers for breast cancer diagnosis. Even though blood has been the main source for biomarker discovery, a number of very promising biomarkers have been identified from other body fluids including urine, sweat, tears, breath, and NAF. In theory, NAF may be the best liquid source for developing a screening diagnostic tool for breast cancer. This is because NAF is in close proximity to the disease area [176], and breast cancer-specific molecules should exist at a much higher concentration in NAFs than in other circulating body fluids. Among many types of biomarkers, circulating cell-free tumor DNA and circulating miRNAs, especially EV-enclosed and tumor-associated miRNAs, hold great promise for early breast cancer detection [12]. With technological advances in epigenetics to detect DNA methylation at a large scale, cancers including breast cancer can be accurately detected by a simple analysis of methylation patterns of ctDNA through a blood test [26,27,38,39]. Although the current ctDNA methylation signatures do not work well for breast cancer detection, a tailored algorithm specifically developed for breast cancer using breast-derived ctDNA may increase the sensitivity for breast cancer detection [40,191,192]. Of note, breath tests have demonstrated the highest potential to be used in clinics given that a rapid breath test has already been shown to accurately predict the risk of breast cancer and abnormal mammograms in women with breast-related symptoms [156,157,158]. We envision that the breath test, combined with mammography, will significantly reduce the false-positive and false-negative diagnosis of mammograms. Breath tests are non-invasive, easy to use, and could potentially detect cancer at a relatively early stage, and they are also suitable for large-scale population screening [163].

It takes time to develop a clinical biomarker. The process of development of a biomarker for early cancer detection is normally divided into five phases [193]. Although many potential non-invasive biomarkers have been reported, most of them remain in phase 1 (preclinical exploratory) or 2 (clinical assay and validation), a few in phase 3 (retrospective longitudinal) or 4 (prospective screening), and none in phase 5 (cancer control). A phase 2 clinical trial to evaluate the use of protein signature in tears for the detection of breast and other cancers was completed (www.clinicaltrials.gov NCT00574678, https://clinicaltrials.gov/ct2/show/NCT00574678) [194]. Another prospective clinical trial to validate the breath test for breast cancer detection was completed (NCT02888366, https://clinicaltrials.gov/ct2/show/NCT02888366) [195] and the results are very promising [49]. A prospective clinical trial, the ASCEND study (NCT04213326, https://clinicaltrials.gov/ct2/show/NCT04213326), is under way to evaluate the CancerSEEK test for multiple cancer detection [196]. GRAIL Inc. has been very active in developing a ctDNA methylation-based blood test for early detection of multiple cancers, including breast cancer. They have already completed two clinical trials, the CCGA case–control study (NCT02889978, https://clinicaltrials.gov/ct2/show/NCT02889978) [197,198] and the STRIVE cohort study (NCT03085888, https://clinicaltrials.gov/ct2/show/NCT03085888) [26,199]. The results obtained from these two studies are being used to conduct two real-world prospective cohort studies, the UK SUMMIT study (NCT03934866, https://clinicaltrials.gov/ct2/show/NCT03934866) [200] and the US PATHFINDER study (NCT04241796, https://clinicaltrials.gov/ct2/show/NCT04241796) [201].

A series of white papers on bioanalysis have been published by the US FDA to guide analytical validation of potential biomarkers. Analytical validity of any biomarkers has to be evaluated for accuracy, reproducibility, and reliability before they become a clinical utility [202,203]. For the clinical application of biomarkers, the standardization of specimen collection, handling, storage, and analysis is key for quality assurance, reliability and reproducibility. With the significant advances in analytical techniques and tumor biology [204], there is a great possibility of a more accurate, sensitive, and cost-effective non-invasive test for early detection of breast cancer being developed in the near future.

## Figures and Tables

**Table 1:** Summary of potential non-invasive biomarkers for early detection of breast cancer.

Sources	Types	Biomarkers Measured	Detection Types (Sample Size)	Sensitivity	Specificity	Notes	References
**Peripheral blood**	Circulating carcinoma proteins	serum proteins: CEA, FASL, OPN, VEGFC, VEGFD, HGF.	breast cancer (100) vs. non-breast cancer subjects (110).	74.7%.	77.0%.	AUC*: 0.79.	[15]
		tumor-associated autoantibodies: FRS3, RAC3, HOXD1, GPR157, ZMYM6, EIF3E, CSNK1E, ZNF510, BMX, SF3A1, SOX2	breast cancer (100) vs. non-breast cancer subjects (110)	72.2%	70.8%	AUC: 0.77	
		serum proteins and tumor-associated autoantibodies: FASL, IL6, IL8, OPN, VEGFD, HGF, FRS3, MYOZ2, RAC3GPR157, ZMYM6, EIF3E, CSNK1E, ZNF510, BMXSF3A1, SOX2	breast cancer (100) vs. non-breast cancer subjects (110)	81.0%	78.8%	AUC: 0.89	
		Videssa Breast consisting of serum proteins: AFP, CA19-9, CEA, TNF-α, VEGF-C, ErbB2 (HER2); and tumor-associated autoantibodies: ANXA1, ATF3, ATP6AP1, BAT4 (GPANK1), BDNF, CTBP1, DBT, HOXD1, IGF2BP1, IGFBP2, ErbB2 (HER2)	women aged 25–75 (1145)	93.0%	64.0%	negative predictive value: 98.0%	[16]
		Videssa Breast consisting of 11 serum protein biomarkers and 33 tumor-associated autoantibodies	women aged under 50 years (545): Breast cancer (32) vs. benign breast tumor (513)	87.5%	83.8%	negative predictive value: 99.1%	[17]
	Circulating tumor cells	CellSearch^®^ system	metastatic breast cancer (55)	47%	N/A	overall positive agreement for both assays: 73% (CTC ≥ 2) and 69% (CTC ≥ 5)	[18]
		AdnaTest^®^ assay	metastatic breast cancer (55)	53%	N/A		
	Circulating cell-free tumor DNA	ctDNA concentrations (≥0.75%)	colorectal and breast cancer patients (10) vs. healthy subjects (10)	>90.0%	>99.0%		[19]
		ctDNA levels (amplifiable per ml of plasma) with CA15-3 expression level	breast cancer (27)	96.0%	N/A		[20]
		ctDNA levels with CTC numbers	breast cancer (30)	97.0%	N/A		
		ctDNA levels with PIK3CA E545K and H1047R mutations	early-stage breast cancer (29)	93.3%	100.0%	accuracy: 96.7%	[21]
		SCGB3A1 DNA methylation in cfDNA	breast cancer (108) vs. female asymptomatic controls (103)	16.8%	80.0%	accuracy: 53.0%	[22,23]
		methylation patterns of six genes in cfDNA: SFN, P16, hMLH1, HOXD13, PCDHGB7, and RASSF1a	breast cancer (125) vs. healthy subjects (104)	79.6%	72.4%	AUC: 0.727	[24]
		methylation analysis of a panel of 16 genes: 12 novel epigenetic markers: JAK3, RASGRF1, CPXM1, SHF, DNM3, CAV2, HOXA10, B3GNT5, ST3GAL6, DACH1, P2RX3, and chr8:23572595; and four internal control markers: CREM, GLYATL3, ELMOD3, and KLF9	breast cancer (87) vs. healthy subjects (80)	86.2%	82.7%		[25]
		GRAIL DNA methylation patterns of cfDNA for detecting various types of cancers including breast cancer	training set: 844	N/A	99.8%	false-positive rate: <1%	[26]
			validation set: 359	N/A	99.3%	false-positive rate: <1%	
		GRAIL DNA methylation patterns of cfDNA for detecting breast cancer	training set: Breast cancer (247)	N/A	N/A	precision: 96% (82/85)	
			validation set: Breast cancer (104)	N/A	N/A	precision: 93% (40/43)	
		DELFI fragmentation patterns of cfDNA	breast cancer (54) vs. healthy subjects (245)	57.0%	98.0%		[27]
		DELFI fragmentation patterns of cfDNA combined with mutations of cfDNA	breast cancer (54) vs. healthy subjects (245)	65.0%	98.0%		
	Circulating miRNAs	a panel of five miRNAs: miR-1246, miR-1307-3p, miR-4634, miR-6861-5p, and miR-6875-5p	breast cancer (1206), non-cancer controls (1343), benign breast diseases (54)	97.3%	82.9%	accuracy: 89.7%	[28]
		a panel of seven miRNAs: Hsa-miR-126-5p, hsa-miR-144-5p, hsa-miR-144-3p, hsa-miR-301a-3p, hsa-miR- 126-3p, hsa-miR-101-3p, and hsa-miR-664b-5p	triple-negative breast cancer (21) vs. healthy subjects (21)	83.8%	74.2%	accuracy: 79.0% AUC: 0.814	[29]
	Extracellular vesicles	fibronectin	breast cancer (240) vs. non-cancer individuals (205)	65.1%	83.2%	AUC: 0.81	[30]
		developmental endothelial locus-1 protein (Del-1)	breast cancer (100) vs. benign breast tumor (38), noncancerous diseases (58), and healthy subjects (46)	92.31%	86.62%		[31]
	Metabolites	seven metabolites in the plasma: Glu, Orn, Thr, Trp, Met-SO, C2, and C3.	training cohort: breast cancer (80) vs. healthy subjects (100) validation cohort: breast cancer (109) vs. healthy subjects (50)	N/A	N/A	AUC in training cohort: 0.87; AUC in validation cohort: 0.80	[32]
		three metabolites (8-hydroxy-2ʹ-deoxyguanosine, 1-methylguanosine, 1-methyl adenosine) combined with CA15-3	malignant breast cancer (120) vs. benign breast disease (47) and healthy subjects (55)	88.8%	86.8%	AUC: 0.94	[33]
		seven metabolites in serum: Dimethyldodecane, galactose, α-glyceryl stearate, methyl stearate, 1(1-methoxycarbonyethyl)-4-(2-methyl-2-trimethylsilyl-oxypropyl) benzene, tetradecane, glucopyranoside	pre-operative breast cancer patients (152) vs. healthy subjects (155)	96%	100%		[34]
		four plasma metabolites: L-octanoylcarnitine, 5-oxoproline, hypoxanthine, docosahexaenoic acid	discovery set: Breast cancer (40) vs. healthy subjects (30); validation set: Breast cancer (30) vs. healthy subjects (16)	N/A	N/A	positive predictive value: 100.0%	[35]
		metabolomics signature in the plasma	breast cancer (91) vs. healthy subjects (20)	up to 100.0%	up to 100.0%		[36]
	Lipids	a panel of five serum free fatty acids: C16:1, C18:3, C18:2, C20:4, C22:6.	breast cancer (140) vs. healthy subjects (202)	83.3%	87.1%	AUC: 0.953	[37]
	Multi-analyte tests	CancerSEEK testing of eight circulating proteins: CA-125, CA19-9, CEA, HGF, myeloperoxidase, OPN, prolactin, and TIMP-1; and mutations in 16 genes in cfDNA in the blood: NRAS, HRAS, KRAS, CTNNB1, PIK3CA, FBXW7, APC, EGFR, BRAF, CDKN2A, PTEN, FGFR2, AKT1, TP53, PPP2R1A, and GNAS	patients with one of the eight cancer types including breast cancer (1005) vs. individuals without known cancers (812)	70.0%	>99%		[38]
			breast cancer (209) vs. healthy subjects (812)	33.0%	>99.0%		
		CancerSEEK tests for different types of cancers	patients with cancers including breast cancer (96) vs. subjects without cancers (9,815)	27.1%	98.9%	positive predictive value: 19.4%	[39]
		CancerSEEK tests for breast cancer	breast cancer (27)	3.7%	N/A		
		CancerSEEK remodeling with CancerA1DE method	first dataset: 1817 patient blood test records second dataset: 626 patient blood test records	70.0%	99.0%	remodeling of the CancerSEEK dataset to improve the sensitivity	[40]
**Other body fluids**	Urine	miRNAs (miR-21, miR-34a, miR-125b, miR-155, miR-195, miR-200b, miR-200c, miR-375, miR-451)	breast cancer (24) vs. healthy subjects (24)	91.7%	91.7%	AUC: 0.932	[41]
		four urinary microRNA types (miR-424, miR-423, miR-660, and let7-i)	breast cancer (69) vs. healthy subjects (40)	98.6%	100.0%		[42]
		miRNA-21 and MMP-1	breast cancer (22) vs. healthy subjects (26)	95.0%	79.0%		[43]
		succinic acid and dimethyl-heptanoylcarnitine	breast cancer (31) vs. healthy subjects (29)	93.5%	86.2%		[44]
	Breath	a set of VOCs of oxidative stress	breast cancer (51) vs. healthy subjects (42)	94.1%	73.8%		[45]
		3-methylhexane	breast cancer (10) vs. healthy subjects (10)	100%	40%		[46]
		decene		100%	40%		
		caryophyllene		100%	60%		
		naphthalene		90%	70%		
		trichlorethylene		80%	70%		
		five breath VOCs: 2-propanol, 2,3-dihydro-1-phenyl-4(1H)-quinazolinone,1-phenyl-ethanone, heptanal and isopropyl myristate	breast cancer (51) vs. healthy subjects (42)	93.8%	84.6%		[47]
		two commercial electronic noses	breast cancer (48) vs. healthy subjects (45)	N/A	N/A	an average of 95% accuracy	[48]
		BreathLink™ point-of-care breath testing systems	breast cancer (50) vs. non-breast cancer subjects (543)	82.0%	77.1%	accuracy: 83%	[49]
		VOCs collected by ultra-clean breath collection balloons	breast cancer (54) vs. non-breast cancer subjects (124)	N/A	N/A	low DF values: Negative predictive value > 99.9%; high DF values: Positive predictive value rising to 100%	[50]
	NAF	Thomsen–Freidenreich (TF) antigen and its biosynthetic precursor Tn antigen	breast cancer (25) vs. healthy subjects (25)	N/A	N/A	detected in 92% of the cancerous breast NAF samples	[51]
		TF, Tn, and age information	breast cancer (83) vs. benign disease (41)	N/A	N/A	AUC: 0.83	[52]
		combination of TF and uPA	breast cancer (83) vs. benign disease (41)	N/A	N/A	accuracy: 84-92%	[53]
		combination of TF, uPA and PAI-1	breast cancer (83) vs. benign disease (41)	N/A	N/A	predictive ability reached 100%	
		deglycase DJ-1 protein	136 patients with nipple discharge (benign: 63; malignant: 73)	75.0%	85.9%		[54]
		proteomic profiles of NAFs	breast cancer (18) vs. healthy subjects (4)	N/A	N/A	39 proteins differentially expressed in tumor-bearing vs. disease-free breasts	[55]
		dehydroepiandrosterone (DHEA) concentration	breast cancer (160) vs. healthy subjects (157)	N/A	N/A	higher DHEA concentrations in NAFs were associated with breast cancer	[56]
	Tears	proteomic profiles of tears	breast cancer (10) vs. healthy subjects (10)	~90.0%	~90.0%		[57]
		a panel of 20 proteins in tears	breast cancer (50) vs. healthy subjects (50)	~70.0%	~70.0%		[58]
		MALDI-TOF-TOF-MS-driven semi-quantitative comparison of tear protein levels	breast cancer (25) vs. healthy subjects (25)	N/A	N/A	more than 20 proteins were differentially expressed in the tears	[59]
		surface-enhanced Raman scattering (SERS) spectra of tear fluid	breast cancer (5) vs. healthy subjects (5)	92%	100%	accuracy: 96%	[60]
	**Apocrine sweat**	**20 sweat markers**	**breast cancer (70) vs. healthy subjects (53)**	**97%**	**72%**		**[61]**

* AUC, area under the receiver operating characteristic (ROC) curve; CTC, circulating tumor cell; cfDNA, cell-free DNA; ctDNA, circulating tumor DNA; DELFI, DNA evaluation of fragments for early interception; MALDI-TOF-TOF-MS, matrix-assisted laser desorption/ionization time-of-flight/time-of-flight mass spectrometry; NAF, nipple aspirate fluid; uPA, urinary plasminogen activator; PAI-1, plasminogen activator inhibitor-1; VOC, volatile organic compound.
